# *Mycoplasma ovipneumoniae* induces sheep airway epithelial cell apoptosis through an ERK signalling-mediated mitochondria pathway

**DOI:** 10.1186/s12866-016-0842-0

**Published:** 2016-09-23

**Authors:** Yanan Li, Zhongjia Jiang, Di Xue, Guangcun Deng, Min Li, Xiaoming Liu, Yujiong Wang

**Affiliations:** 1 Key Laboratory of Ministry of Education for Conservation and Utilization of Special Biological Resources in the Western, Yinchuan, Ningxia 750021 China; 2 College of Life Science, Ningxia University, Yinchuan, Ningxia 750021 China; 3 Ningxia Key Laboratory of Clinical and Pathogenic Microbiology, the General Hospital of Ningxia Medical University, Yinchuan, Ningxia 750004 China

**Keywords:** *M. ovipneumoniae*, Sheep, Airway epithelial cells, Apoptosis, Mitochondrial pathway

## Abstract

**Background:**

*Mycoplasma ovipneumoniae* (*M. ovipneumoniae*) is a species of Mycoplasma bacteria that specifically infects sheep and goat, causing ovine infectious pleuropneumonia. However, the mechanism underlying the pathogen-host interaction between *M. ovipneumoniae* and airway epithelial cells is unknown.

**Methods:**

A primary air-liquid interface (ALI) epithelial culture model generated from the bronchial epithelial cells of Ningxia *Tan sheep* (*ovis aries*) was employed to explore the potential mechanism of *M. ovipneumoniae*-induced cell apoptosis by characterizing the production of reactive oxygen species (ROS), methane dicarboxylic aldehyde (MDA) and anti-oxidative enzymes, as well as the mitochondrial membrane potentials, cytochrome C release, and activities of ERK and caspase signalling pathways.

**Results:**

Increased ROS production and MDA concentration with mitochondrial membrane dysfunction and apoptotic cell death but decreased expression of the antioxidant enzymes catalase (CAT), glutathione synthetase (GSS), total superoxide dismutaes (T-SOD) and Mn-SOD were observed in sheep airway epithelial cells infected with *M. ovipneumoniae*. Mechanistically, the *M. ovipneumoniae*-induced cell apoptosis and disruption of mitochondrial integrity reflected mechanisms by which pathogen-activated mitogen-activated protein kinase (MAPK) signalling sequentially led to mitochondrial damage and release of Cyt-C into the cytoplasm, which in turn triggered the activation of caspase signalling cascade, resulting in the apoptosis of host cells.

**Conclusions:**

These results suggest that *M. ovipneumoniae*-induced ROS and MAPK signalling-mediated mitochondrial apoptotic pathways might play key roles in the pathogenesis of *M. ovipneumoniae* infection in sheep lungs.

**Electronic supplementary material:**

The online version of this article (doi:10.1186/s12866-016-0842-0) contains supplementary material, which is available to authorized users.

## Background

*Mycoplasma pneumonia* (*M. ovipneumoniae*) is a species of mycoplasma bacteria that specifically infect both sheep (*Ovis aries*) and goats. Since these bacteria were first isolated from the lung tissue of sheep with lung adenoma in 1963 [1], the mechanisms underpinning its pathogenesis have been extensively investigated. Studies have been demonstrated that airway epithelial cells are the main targets of *M. ovipneumoniae* infections and that these cells play an important role in host-pathogen interactions and the pathogenesis of mycoplasma infections in the lung, beyond their roles as the first line of physical barriers in defending against microbial infections and environmental stresses [2, 3]. In this context, airway epithelial cells maintain local immune homeostasis by producing cytokines and mucoproteins [4]. In mycoplasma infections, the adhesion of the pathogens to airway epithelial cells is the first key step towards infection, through which mycoplasma bacteria gain the ability to escape from clearance host immune responses [5].

A compelling body of evidence suggests that the metabolic products of mycoplasma cells induce significant oxidative damage, cell pathological changes and apoptosis by producing a large amount of H_2_O_2_ after they adhered to host epithelial cells [6–12]. Under physiological conditions, the host cells can balance the metabolism of oxygen-free radicals through defence mechanisms [13]. However, under pathological circumstances, oxidative stress caused by excessive oxygen free-radicals might lead to cell injury by mechanisms involved in mitochondrial dysfunction [14, 15] and the reduction of activities of antioxidant enzymes, including superoxide dismutase (SOD) [16], catalase (CAT) [17, 18] and glutathione synthetase (GSS) [19, 20]. The increased production of reactive oxygen species (ROS) [21–23] and methane dicarboxylic aldehyde (MDA) [24] are often accompanied with oxidative stress. Thus, a disruption of various signal transduction pathways is the main underlying mechanism of cell injury [25–29]. Among these signalling pathways, mitogen-activated protein kinase (MAPK)/extracellular signal-regulated kinase (ERK) signalling is a well-studied pathway involving the regulation of oxidative stress-induced cell apoptosis and cell damage [30, 31].

ERK is a member of the mitogen-activated protein kinases (MAPKs) signalling cascade families, which includes the ERK1 and ERK2 subunits, with respective to the molecular weights of 44 and 42 kD [32]. ERK1 and ERK2 share 90 % homology and use the same substrate in vitro. These enzymes can be activated through phosphorylation by different extracellular irritants, such as mitogen, growth factors and oxidative stress [33]. The ERK signalling pathway plays a key role in the regulation of multiple cell functions, including cell proliferation, survival, apoptosis and migration [34]. In addition, several lines of evidence have suggested that the ERK signalling pathway could be activated in response to cell damage by oxidative stress in airway epithelial cells [35–37]. Mechanistically, oxygen-free radicals induce mitochondrial damage, accompanied with a release of cytochrome C (Cyt-C) into the cytoplasm, in which Cyt-C activates caspases, such as caspase-9 and caspase-3, eventually promoting cell apoptosis [38–40]. However, the BCL-2 family members are mitochondrial membrane anti-apoptotic proteins involved in the transformation of the mitochondria transmembrane potential [41]. The main anti-apoptotic proteins of BCL-2 family, such as Bcl-2 and Bcl-xl, inhibit the release of Cyt-C and protect cells from apoptosis by inhibiting the activation of caspases acting as downstream signals of Cyt-C. Notably, the activation of pro-apoptotic proteins also damages the structure and function of mitochondria [42]. Cell apoptosis could be induced by decreasing the expression and inactivation of ERK1/2, and by causing alterations in the expression of apoptosis-related genes. For example, an increased expression and activation of ERK1/2 delays the onset of apoptosis and increases the expression of Bcl-xl [43]. In contrast, the inhibition of ERK1/2 activity and expression could down-regulate the expression of the anti-apoptotic homologues Bcl-2 and Bcl-xl, although there is no effect on the expression of the pro-apoptotic protein Bak [44].

These results suggest that pathogen-induced oxidative stress is key for the pathogenesis of mycoplasma infection. Thus, we hypothesized that MAPK/ERK signalling might be involved in the cell death induced by *M. ovipneumoniae* infection in sheep airway epithelial cells. Therefore, we tested this hypothesis and examined the pathogen-host interaction of *M. ovipneumoniae* cells and normal sheep bronchial airway epithelial cells using an air-liquid interface (ALI) culture model. The results showed that an *M. ovipneumoniae* infection could induce oxidative stress and mitochondrial dysfunction in part through the MAPK/ERK signalling pathway in sheep airway epithelial cells.

## Results

### The cell death and mitochondrial dysfunction of sheep airway epithelial cells induced by *M. ovipneumoniae* infection

**Fig. 1:**
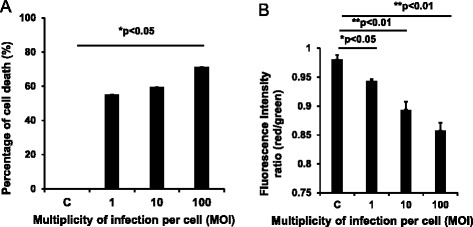
Impact of M. *ovipneumoniae* infection on the cell death and mitochondrial membrane potential of sheep airway epithelial cells. Sheep airway epithelial cells cultured on an air-liquid interface model were apically infected with 1, 10 and 100 MOI of *M. ovipneumoniae* (MO) at 37 °C for 24 h. The cell viability was detected in terms of a LDH assay (**a**) and the mitochondrial membrane potential was determined using a potential-sensing fluorescent probe (JC-1) (**b**). **a** The percentage of cell death of ALI sheep airway epithelial cells infected with the indicated doses of MO, and a dose-dependent cell death was induced by MO infections. **b** Mitochondrial membrane potential (ΔΨm) of airway epithelial cells infected with indicated dose of MO, and the fluorescence intensity of both mitochondrial JC-1 monomers (λex 514 nm, λem 529 nm) and aggregates (λex 585 nm, λem 590 nm) were measured. The mitochondrial ΔΨm of airway epithelial cells were calculated as the fluorescence ratio of red over green. The mitochondrial ΔΨm decreased with increasing MOI of infection. Data were expressed as the means ± SD of three independent experiments, and each experiment had six replicated ALI cultures (*N* = 18). Compared with the uninfected controls, *: *p* < 0.05; **: *p* < 0.01. The cell numbers of each transwell with diameter of 24 mm was determined as 10^7^

Upon cell death and plasma membrane damage, lactate dehydrogenase (LDH) is rapidly released into the cell culture medium, therefore accessing the free LDH used to quantify cell death in response to *M. ovipneumoniae* infection [45]. Compared with the uninfected control, the dose-dependent cytotoxicity of sheep airway epithelial cells was observed in response to *M. ovipneumoniae*. Consistent with our previous findings [27], 71.26 % of cell death was determined in sheep ALI airway epithelial cells infected by *M. ovipneumoniae* at an MOI of 100 (Fig. 1a). Importantly, the increased dose-dependent cell death was inversely correlated with a decreased mitochondrial membrane potential in *M. ovipneumoniae-*infected sheep airway epithelial cells based on the results of the JC-1 assay, which assesses the integrity of the mitochondrial membrane (Fig. 1b). JC-1 is a fluorescent probe for testing mitochondrial membrane potential. Compared with uninfected control cells, the fluorescent intensity ratios (red/green) of JC-1 in airway epithelial cells were decreased 3.76, 8.85 and 12.55 %, with increased M. *ovipneumoniae* infection at an MOI of 1, 10 and 100, respectively (Fig. 1b). Mechanistically, a decline in the mitochondrial membrane potential has been suggested as a symbolic event during early periods of apoptosis [46].

### Effect of *M. ovipneumoniae* infection on the production of MDA and SOD

**Fig. 2:**
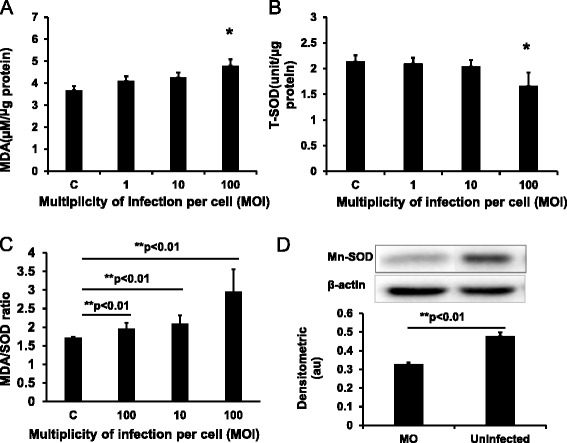
Effects of *M. ovipneumoniae* infections on the production of MDA and SOD in sheep airway epithelial cells. Sheep airway epithelial cells cultured on an air-liquid interface model were apically infected with 1, 10 and 100 MOI of *M. ovipneumoniae* (MO) at 37 °C for 24 h, the levels of MDA and SOD were measured using appropriate kits. **a** A dose-dependent increase of MDA production was observed in cells infected with MO. **b** A dose-dependent decrease of total-SOD (T-SOD) level of airway epithelial cells was induced by the MO infection. **c** A significant increase in the MDA/SOD ratio in sheep airway epithelial cells infected with MO. **d** A representative image of immunoblot showing the reduced expression of Mn-SOD protein in ALI cultures infected with MO (MOI = 100) (*top panel*) compared with the uninfected cells, which was significantly different as determined based on the value of densitometric arbitrary units (A.U.) calculated as the densitometric signal of Mn-SOD protein over that of the corresponding β-actin internal control (*bottom panel*). Data were expressed as the means ± SD from three independent experiments, and each experimental had six replicated ALI cultures (*N* = 18) in **a**–**c**. Compared with the uninfected controls, *: *p* < 0.05; **: *p* < 0.01. The cell numbers of each transwell with diameter of 24 mm was determined as 10^7^

We next investigated whether the observed mitochondrial dysfunction resulted from oxidative stress induced by *M. ovipneumoniae* infection. To this end, we examined the production of MDA and SOD, using MDA as an indicator of lipid peroxidation and a marker for oxidative stress. SOD is an important antioxidant enzyme that induces disproportionation by catalysing the superoxide anion, which could serve as an anti-oxidative marker. As expected, *M. ovipneumoniae* infection induced MDA generation and markedly increased MDA production by 30.2 % in cells infected with *M. ovipneumoniae* at an MOI of 100 compared with the uninfected control (Fig. 2a), suggesting that *M. ovipneumoniae* infection induced lipid peroxidation in sheep airway epithelial cells. In contrast, *M. ovipneumoniae* infection showed a dose-dependent decrease in SOD activity and expression (Fig. 2b). Compared with the control, *M. ovipneumoniae* infection at an MOI of 100 reduced the total SOD (T-SOD) activity by 22.6 % (Fig. 2b). In addition, the MDA/SOD ratio, an index of oxidative stress, was also dramatically increased with infection in a dose-dependent manner (Fig. 2c). Because Mn-SOD is a major antioxidant enzyme of T-SOD, distributed primarily throughout the mitochondrial matrix [47], the expression of Mn-SOD was also examined. Consistent with this notion, the Mn-SOD protein was significantly reduced in sheep airway epithelial cells following M. *ovipneumoniae* infection (Fig. 2d). These data suggest that *M. ovipneumoniae* can induce lipid peroxidation and oxidative stress in sheep airway epithelial cells.

### The effect of *M. ovipneumoniae* infection on the production of CAT and GSS

**Fig. 3:**
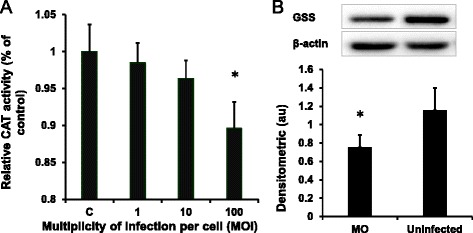
Effects of *M. ovipneumoniae* infections on CAT activity and GSS expression. Sheep airway epithelial cells cultured on an air-liquid interface model were apically infected with 1, 10 and 100 MOI of *M. ovipneumoniae* (MO) at 37 °C for 24 h, and the activities of CAT and GSS expression were determined. **a** A dose-dependent decrease of CAT activity was observed in airway epithelial cells infected with MO. **b** A representative image of immunoblot showed the reduced expression of GSS protein in ALI cells infected with MO (MOI = 100) (*top panel*) compared with the uninfected cells, which was significantly different based on the value of densitometric arbitrary units (A.U.), which was calculated based on densitometric signal of GSS protein over that of the corresponding β-actin internal control (*bottom panel*). Data were expressed as the means ± SD from three independent experiments, with each experimental had six replicated ALI cultures (*N* = 18) in A. Compared with the uninfected controls, *: *p* < 0.05; **: *p* < 0.01. The cell numbers of each transwell with diameter of 24 mm was determined as 10^7^

Increased cellular ROS levels can damage proteins, lipids, and nucleic acids and reduce the activities of various antioxidant enzymes, such as CAT. Hence, we determined the activities of CAT in airway epithelial cells treated with *M. ovipneumoniae*. Compared with uninfected cells, a 10.4 % decrease in CAT activity was observed in airway epithelial cells infected with *M. ovipneumoniae* at an MOI of 100 (Fig. 3a). Moreover, glutathione synthetase (GSS), another antioxidant enzyme, was reduced in response to the increased ROS concentration. As expected, these results showed that the expression of GSS protein was significantly reduced in epithelial cells following an *M. ovipneumoniae* infection (Fig. 3b).

### RAS/MEK/ERK is involved in sheep airway epithelial cells in response to *M. ovipneumoniae* infection

**Fig. 4:**
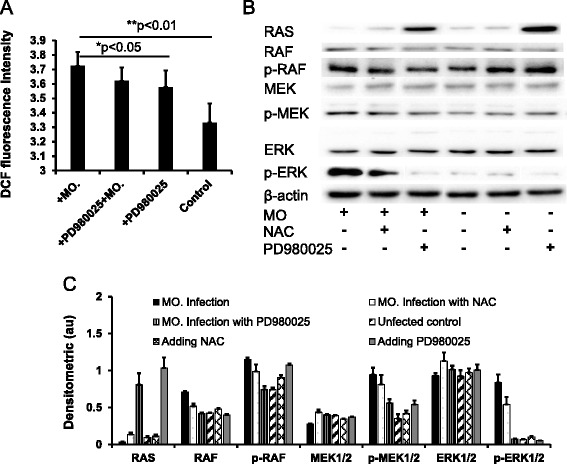
The production of ROS and expression of MEK/ERK signalling in sheep airway epithelial cells induced by M. *ovipneumoniae* infection. Sheep airway epithelial cells cultured on an air-liquid interface model were apically infected with *M. ovipneumoniae* (MO) at an MOI of 100 for 24 h, and the production of ROS was measured by a fluorometrical assay using DCFH-DA dye (**a**), and changes in the expression of key molecules of the MEK/ERK signalling pathway was assessed using an immunoblotting method. **a** An infection of MO led an increased production of ROS in airway epithelial cells, which could be partially inhibited by MEK/ERK signalling inhibitor PD980025. **b** An immunoblotting assay revealed an evoked expression of RAS, RAF, p-RAF, MEK, p-MEK, ERK and p-ERK proteins in cells infected with MO, and this induction was inhibited PD980025 and ROS scavenger NAC. **c** Semi-quantitative analysis of the expression of proteins in (**b**) by evaluating the relative densitometric densities using arbitrary units (A.U.), calculated according to the densitometric signal of a protein of interest over that of the corresponding β-actin internal control. Data were expressed as the mean ± SD from three independent experiments. Compared to the uninfected controls, *: *p* < 0.05; **: *p* < 0.01 in cells untreated with PD980025. The cell numbers of each transwell with diameter of 24 mm was determined as 10^7^

Accumulating evidence has shown that *M. ovipneumoniae* induces oxidative stress in airway epithelial cells. Previous studies have revealed that increased ROS production could induce oxidative stress [48] and impact the regulation of the RAS/MEK/ERK signalling pathway [49]. Thus, we next examined whether the ERK signalling was involved in *M. ovipneumoniae*-induced ROS generation. The airway epithelial cells were infected with *M. ovipneumoniae* at an MOI of 100 in the presence or absence of PD980025 (an ERK inhibitor). The ROS levels were detected using 2′,7′-dichlorofluorescein diacetate (DCFH-DA). The results showed that *M. ovipneumoniae* infection significantly increased ROS production in ALI cultures, while PD980025 markedly inhibited M. *ovipneumoniae*-induced ROS production (Fig. 4a), suggesting that the ERK signalling was involved in M. *ovipneumoniae*-induced ROS generation. In order to further understand the underlying mechanism of RAS/MEK/ERK signalling in airway epithelial cells in response to *M. ovipneumoniae* infection, an immunoblotting assay was employed to analyse the RAS, RAF, phosphorylated-RAF (p-RAF), MEK, p-MEK, ERK and p-ERK proteins in airway epithelial cells in the presence or absence of NAC (a ROS scavenger) or PD980025 (Fig. 4b and c). The results showed that *M. ovipneumoniae* could activate the phosphorylation of RAS, MEK and ERK, while the presence of NAC or PD980025 remarkably inhibited the activation of phosphorylated RAS, MEK and ERK. These data clearly suggest that the activation of the RAS/MEK/ERK signalling pathway plays a major role in sheep airway epithelial cells in response to *M. ovipneumoniae* infection, which also impacts the increased ROS production in sheep airway epithelial cells following M. *ovipneumoniae* infection.

### Sheep airway epithelial cell apoptosis induced by *M. ovipneumoniae* infection

**Fig. 5:**
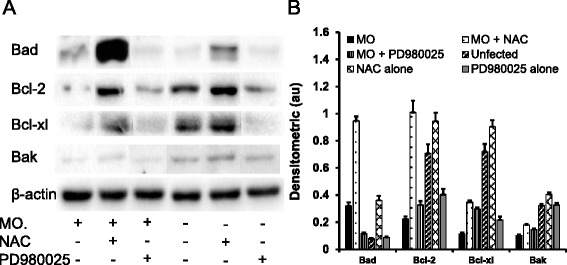
Induction of the expression of BCL-2 family in sheep airway epithelial cells infected with M. *ovipneumoniae.* Sheep airway epithelial cells cultured on an air-liquid interface model were apically infected with *M. ovipneumoniae* (MO) at an MOI of 100 for 24 h, changes in the expression of BCL-2 family members was assessed using an immunoblotting assay. **a** Representative images of immunoblots of indicated proteins, revealed an evoked expression of pro-apoptotic proteins Bad, Bcl-2, Bcl-xl and Bak, and the MO-induced expression of these proteins could be diminished by MEK/ERK signalling PD980025 or ROS scavenger NAC. **b** Semi-quantitative analysis of the expression of proteins in (**a**) by evaluating relative densitometric densities using arbitrary units (A.U.), calculated based on the densitometric signal of a protein of interest over that of the corresponding β-actin internal control

In order to further reveal the potential molecular mechanism underlying the mitochondria damage of sheep airway epithelial cells induced by *M. ovipneumoniae* infection, the changes in BCL-2 family proteins and downstream Cyt-C and caspase signalling cascades were evaluated by an immunoblotting assay [41, 42]. The immunoblots showed that *M. ovipneumoniae* infections significantly inhibited the expression of Bcl-2, Bcl-xl and Bak but that it did not alter the expression of Bad (Fig. 5a and b). Interestingly, the addition of the ROS scavenger NAC significantly increased Bcl-2 and Bcl-xl protein levels, regardless of M. *ovipneumoniae* infection, suggesting that the *M. ovipneumoniae*-inhibited the expression of BCL-2 family members in part by producing ROS in airway epithelial cells (Fig. 5a and b). In addition, BCL-2 family members are downstream genes of the ERK signalling pathway; thus, we examined the effect of *M. ovipneumoniae*-activated phosphorylated ERK on the expression of BCL-2 family proteins. Interestingly, the presence of the ERK inhibitor PD980025 restored the expression of BCL-2 family members following *M. ovipneumoniae* infection in sheep airway epithelial cells (Fig. 5a and b), indicating that *M. ovipneumoniae* induces mitochondrial dysfunction by inhibiting the anti-apoptosis proteins, Bcl-2 and Bcl-xl, through mechanisms involving ROS production and the ERK signalling pathway.

**Fig. 6:**
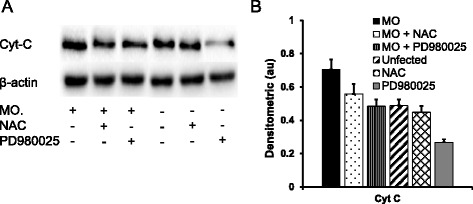
Induction of the expression of Cyt-c in sheep airway epithelial cells infected with M. *ovipneumoniae*. Sheep airway epithelial cells cultured on an air-liquid interface model were apically infected with *M. ovipneumoniae* (MO) at an MOI of 100 for 24 h, changes in the expression of mitochondrial Cyt-c were assessed by an immunoblotting assay. **a** Representative images of immunoblots of Cyt-c and β-actin, revealed an increased release of Cyt-c in cells infected with MO, and the increased level of Cyt-c was reduced in the presence of MEK/ERK signalling PD980025 or ROS scavenger NAC. **b** Semi-quantitative analysis of the expression of proteins in (**a**) by evaluating relative densitometric densities using arbitrary units (A.U.), which was calculated with densitometric signal of a protein of interest over that of the corresponding β-actin internal control

**Fig. 7:**
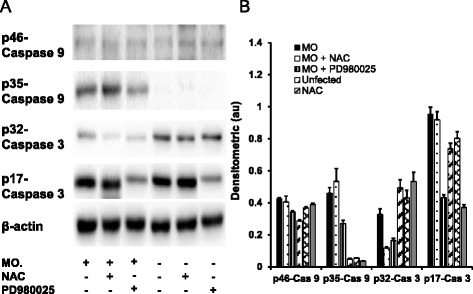
Induction of the expression of caspase-3/9 in sheep airway epithelial cells infected with M. *ovipneumoniae*. Sheep airway epithelial cells cultured on an air-liquid interface model were apically infected with *M. ovipneumoniae* (MO) at an MOI of 100 for 24 h, changes of the expression of caspases was assessed using an immunoblotting assay. **a** Representative images of immunoblots of indicated caspases or their activated forms, revealed an enhanced activities of caspase 3 and 9 in airway epithelial cells infected with MO, and an addition of MEK/ERK signalling PD980025 or ROS scavenger NAC resulted in reducing the MO-induced activities of these caspases. **b** Semi-quantitative analysis of the expression of proteins in (**a**) by evaluating the relative densitometric densities using arbitrary units (A.U.), calculated based on the densitometric signal of a protein of interest over that of the corresponding β-actin internal control

The release of Cyt-C is another hallmark of mitochondrial dysfunction and cell apoptosis [39], immunoblotting analysis also showed a strikingly increased abundance of Cyt-C in airway epithelial cells following *M. ovipneumoniae* infection, and the addition of NAC or PD980025 significantly decreased Cyt-C release (Fig. 6a and b). Consequently, increased cytosol Cyt-C triggers the activation of the caspase cascade and cell apoptosis [40]. Immunoblot analysis showed that *M. ovipneumoniae* infection significantly increased the expression of p35-caspase-9 and p17-caspase-3 and decreased the expression of p32-caspase-3 (Fig. 7a and b). In contrast, the expression of p35-caspase-9 increased, but the expression of p17-caspase-3 and p32-caspase-3 decreased in the presence of ROS scavenger NAC (Fig. 7a and b). Similarly, the decreased expression of p35-caspase-9 and p32-caspase-3 was observed in cells exposed to the ERK inhibitor PD980025 (Fig. 7a and b). These data suggest that *M. ovipneumoniae* infection could induce the release of Cyt-C into the cytoplasm and activate caspase-9 and caspase-3 signalling cascades through mechanisms involving ROS production and ERK signalling pathways.

## Discussion

We previously described MyD88-dependent toll-like receptor (TLR) signalling induced by *M. ovipneumoniae* in sheep airway epithelial cells [27]. In the present study, we examined the underlying mechanism of airway epithelial cell death induced by *M. ovipneumoniae* infection using an ALI culture model. The results showed that *M. ovipneumoniae* infection induces ROS production, cell death and mitochondrial membrane dysfunction and increased MDA concentration but decreased the expression of antioxidant enzymes CAT, GSS, T-SOD and Mn-SOD. Mechanistically, the *M. ovipneumoniae*-induced cell death and mitochondrial dysfunction in part reflected mechanisms by which the pathogen activates RAS/MEK/ERK signalling, leading to mitochondrial damage and Cyt-C release into the cytoplasm, which in turn triggers the activation of the caspase signalling cascade, eventually leading to host cell apoptosis.

In the present study, an ALI culture model generated using primary sheep bronchial epithelial cells was employed to assess the pathogen-host interaction between *M. ovipneumoniae* and airway epithelial cells. The epithelial cells cultured in an ALI status fully differentiated into distinct epithelial cell types and formed a pseudostratified epithelium comprising tight junctions. The apical cell surface represented an environment comparable to the airway lumen in vivo [27, 50]. By using this novel model, the mechanism of *M. ovipneumoniae*-induced oxidative stress in sheep airway epithelial cell was explored. In the present context, *M. ovipneumoniae* induced the generation of ROS and reduced the expression and activity of antioxidant enzymes, including T-SOD, Mn-SOD, CAT and GSS, suggesting that oxidative stress was induced by *M. ovipneumoniae* in sheep airway epithelial cells. For example, Mn-SOD is one of the primary defence substances in mitochondria and is located in the mitochondrial matrix. Notably, reduced Mn-SOD activity is correlated with the lack of mitochondrial defence [47]. Therefore, a decreased mitochondrial membrane potential in airway epithelial cells following the *M. ovipneumoniae* infection suggests that the pathogen induces oxidative stress to injure mitochondria, which sequentially triggers a mechanism of mitochondrial damage. Similarly, the MDA is a marker of lipid peroxide, which reduces member fluidity and induces cell apoptosis [51]. Consistent with this notion, the increased concentration of MDA and ROS was observed in cells infected with *M. ovipneumoniae*, accompanied with the reduced expression and inactivation of antioxidant enzymes, as well as the dysfunction and disruption of the mitochondrial membrane structure in host cells, evidenced by a decreased mitochondrial membrane potential.

The BCL-2 family members are closely related to mitochondrial membrane integrity, of which Bcl-2 and Bcl-xl are anti-apoptotic factors of this family. In the present study, the expression of Bcl-2, Bcl-xl and Bak in sheep airway epithelial cells was inhibited in cells infected by *M. ovipneumoniae*, indicating that an apoptotic event might occur in these infected cells [42, 52]. Bak is an interesting member of the BCL-2 family, which can exert both pro-apoptotic and anti-apoptotic roles in a cell context-dependent manner [53]. The reduced expression of Bak in *M. ovipneumoniae*-infected cells might suggest that this enzyme plays an anti-apoptotic role in the *M. ovipneumoniae*-induced mitochondrial damage mechanism. In order to investigate whether *M. ovipneumoniae*-induced oxidative stress impacts the expression of BCL-2 family proteins, cells infected with *M. ovipneumoniae* were treated with the ROS scavenger NAC. Interestingly, *M. ovipneumoniae-*reduced the expression of Bcl-2 and Bcl-xl proteins in airway epithelial cells, and this expression was restored after the addition of NAC. Notably, *M. ovipneumoniae* infection did not significantly alter the expression of Bad, likely reflecting the location of Bad in epithelial cells, as this protein was not consistently located on mitochondria in a cell type-dependent context and could be activated and translocated to mitochondria [42].

The transformation of the mitochondrial membrane potential can convert the transformation of the membrane permeability [46] through a mechanism involving the release of cytochrome C (Cyt-C) [38] and the activation of the caspase 3/9 cascade [39, 40]. In the present study, the increased release of Cyt-C and activation of caspase3/9 signalling were observed in *M. ovipneumoniae*-infected sheep airway epithelial cells, suggesting that a mitochondria-related signalling pathway was involved in the *M. ovipneumoniae*-induced apoptosis of epithelial cells. Equally noteworthy, the release of Cyt-C and activation of caspase 3/9 reflected *M. ovipneumoniae*-induced oxidative stress, consistent with evidence that the ROS scavenger NAC could suppress Cyt-C release and caspase-3 activation in *M. ovipneumoniae*-infected airway epithelial cells. However, NAC had no effect on caspase-9 activation, consistent with a previous study showing that the activation of caspase-9 was not dependent on the concentration of cytoplasmic Cyt-C [54]. Therefore, these findings suggested that the *M. ovipneumoniae*-induced mitochondrial damage most likely depended on the levels of cytoplasm cytochrome C in airway epithelial cells.

In addition, mitochondrial signalling occurs downstream of the ERK signalling pathway. Therefore, targeting ERK signalling might impact the mitochondria-related apoptotic signalling pathway. Indeed, the inhibition of ERK signalling using PD980025 restored the expression of anti-apoptotic BCL-2 family proteins, and suppressed Cyt-C release and caspase 3/9 activation in *M. ovipneumoniae*-infected sheep airway epithelial cells. Together with evidence that ROS activates ERK signalling and *M. ovipneumoniae* infection activates the RAS/MEK/ERK signalling pathway, these data suggested that the mitochondria signalling pathway was mediated by the RAS/MEK/ERK signalling in sheep airway epithelial cells in response to *M. ovipneumoniae* infections.

Interestingly, glycerol metabolism has been implicated in the generation of H2O2 and ROS in many mycoplasma species [9–11, 55]. However, there are no data concerning the involvement of glycerol metabolism in the production of ROS and H_2_O_2_ for *M. ovipneumoniae*, although the genome sequence of the *M. ovipneumoniae* SC1 strain was completed in 2011 [56]. Thus, the role of glycerol metabolism in *M. ovipneumoniae*-induced oxidative stress warrants further investigation in the future.

## Conclusions

**Fig. 8:**
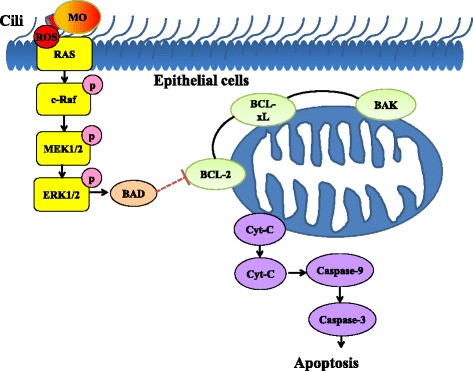
Schematic diagram of an apoptotic cell death induced by M. *ovipneumoniae* infection via signalling pathways converging at mitochondria. An infection of *M. ovipneumoniae* generated ROS triggers the activation of ERK signalling of sheep airway epithelial cells, which induce cell apoptosis through an ERK signalling-mediated mitochondria pathway

In the present study, we attempted to uncover the mechanism underlying the pathogen-host interaction between *M. ovipneumoniae* and sheep airway epithelial cells using an ALI culture model. The results demonstrated a mechanism of cell death-regulated signalling pathways in mitochondria of airway epithelial cells in response to Mycoplasma infections (Fig. 8) [57], by which a *M. ovipneumoniae* infection could induce oxidative stress by increasing production of ROS and lipid peroxidation, and the inhibition of antioxidant enzyme activity in airway epithelial cells. Mechanistically, *M. ovipneumoniae* bacteria activate RAS/MEK/ERK signalling, which disrupted the integrity of mitochondrial membrane through the reduction of the expression of BCL-2 family anti-apoptotic proteins, increasing the release of Cyt-C and activating the caspase signalling cascade, thus sequentially inducing cell apoptosis.

## Methods

### Propagation of *Mycoplasma Ovipneumoniae*

The *M. ovipneumoniae* Queensland Strain Y98 [58] was obtained from the China Institute of Veterinary Drug Control (Beijing, China). As previously described [58], the mycoplasma bacterium was cultured and propagated in a mycoplasma broth containing mycoplasma broth base CM403, supplement-G SR59 (OXOID, Hampshire, UK), 0.5 % glucose, and 0.002 % phenol red at 37 °C in 1 % CO_2_. The titre of *M. ovipneumoniae* culture was determined based on the metabolic activity of the bacterial cells in the medium, presented as a colour change unit (CCU)/mL [59]. The CCU assay is accurate and comparable to traditional colony formation units (CFU) on agar plates for the titration of mycoplasma species, although the results of this assay might provide a higher estimate of cell numbers, consistent with the DNA content of the cell pellet and published genome sizes [60, 61]. Prior to use, the bacterial cells were washed three times in PBS in order to minimize the effects of potential contaminants originating from the culture medium (e.g., LPS or other molecules).

### In vitro ALI culture of sheep bronchial epithelium and infection

The present study was approved by the ethics committee for the use of animals at Ningxia University. The bronchi of Chinese Tan sheep (*O. aries*) (1.5 to 2.0 years old) were obtained from a local slaughterhouse. Despite the need for consent is deemed unnecessary according to national regulations, an informed verbal consent was obtained from the sheep owners. The ethics committee for the use of animals of Ningxia University approved this study. The ALI culture of airway epithelial cells was generated as previously described [27]. Briefly, the bronchus was longitudinally opened after the muscle and vascular tissues were removed and washed in ice-cold 1 % Pen Strep–1.0 g/mL fungizone PBS. Then, the bronchial specimens were incubated in a tube filled with epithelial cell dissociation buffer (DMEM Pen Strep–fungizone medium containing 1.5 mg/mL pronase and 10 μg/mL DNase I) at 4 °C for 24–36 h, with rotation during dissociation. The enzymatic dissociation was terminated by adding FBS at a final concentration of 10 %, and the epithelial cells were then collected by centrifugation at 500 *g* for 10 min at 4 °C as previously described [4]. The cell pellet was re-suspended in DMEM 5 % FBS, followed by incubating the cells on tissue culture plates (Primera; Becton-Dickinson Labware, Franklin Lakes, NJ) for 2–4 h in 5.5 % CO_2_ at 37 °C for the adhesion of fibroblasts, after which the non-adherent cells were collected by centrifugation and re-suspended in Bronchial Epithelial cell Growth Medium (BEGM) (Lonza, Basel, Switzerland). The number of total cells was determined using a haemocytometer counting chamber. In vitro, ALI cultures of sheep bronchial epithelial cells were cultured previously described [50]. Briefly, the polycarbonate/polyester porous (0.4 μM pores) transwell membranes (PCF Millicell inserts, Millipore, Bedford, MA) were pre-coated with 60 μg/mL of type I rat tail collagen (BD Biosciences, San Jose, CA, USA). Approximately 1 × 10^5^ cells were seeded into a 0.6 cm^2^ Millicell insert membrane, and subsequently the inserts were incubated in BEGM medium (containing 5 % FBS) in the apical and basolateral compartment of a 24-well plate at 37 °C in 6 % CO_2_ for approximately 18–24 h. Then, the membranes were washed with pre-warmed PBS to remove unattached cells and re-fed with BEGM medium and cultured for 2 additional days. To establish an air–liquid interface, 2 % Ultroser G (USG) medium (Pall, Port Washington, NY, USA) was added to the basolateral side of the chamber. The medium was refreshed twice a week, and the top of the membrane culture remained visibly dry. The polarized and highly differentiated airway epithelial cell layer was achieved after 2 weeks of ALI culture. For a 2.4-cm diameter Millicell insert membrane, 1 × 10^7^ well-differentiated epithelial cells were determined [50]. A well-differentiated 4-week ALI culture was used for infection. For infection, *M. ovipneumoniae* cells were suspended, diluted in 2 % Ultroser G medium, and applied on the apical surface of ALI epithelial cells for infection at the indicated time periods, and an equal volume of 2 % Ultroser G medium was used as an uninfected control. Volumes of 0.5 and 0.1 mL were employed to cover the wells at diameters of 2.4 and 1.2 cm, respectively.

### LDH assay

Cell death was measured using a lactate dehydrogenase (LDH) assay based on the detection of the LDH released from injured cells. Mechanistically, LDH can oxidize lactate to generate NADH, which in turn reacts with INT, determined as a yellow coloured substrate with maximum absorbance at 450 nm (Beyotime Company, Jiangsu, China). The 6-week ALI cultures were infected at a multiplicity of infection (MOI) of 1, 10 and 100 per *M. ovipneumoniae* cell through the application of bacterial cells on the apical surface of cultures for 24 h and then incubated with LDH staining solution at 37 °C for 1 h. Then, the supernatant was used to examine the relative LDH activity using a Tecan Safire 5 microplate reader (450 nm).

### Mitochondrial membrane potential (MMP) assay

The integrity of the mitochondrial membrane was evaluated based on the mitochondrial membrane potential (MMP) using a JC-1 fluorescent probe. In normal cells, JC-1 exists as a monomer (green) in the cytosol and accumulates as aggregates (red) in mitochondria through the induction of higher MMP. In apoptotic and necrotic cells, JC-1 remains in the monomeric form and stains as green fluorescence in the cytosol. The 6-weeks ALI cultures were infected with MOI of 1, 10 and 100 of *M. ovipneumoniae* by applying the bacterial cells on the apical surface for 24 h and then incubated with JC-1 (Beyotime Company, Jiangsu, China) staining solution (5 μg/mL) at 37 °C for 20 min. The fluorescence intensity of both mitochondrial JC-1 monomers (λ_ex_ 514 nm and λ_em_ 529 nm) and aggregates (λ_ex_ 585 nm and λ_em_ 590 nm) was detected using a Tecan Safire 5 microplate reader. The Δ*Ψ*_m_ of the cells was calculated as the fluorescence ratio of red to green.

### Measurement of MDA and SOD

The MDA concentration was determined using a thiobarbituric acid (TBA) test as previously described [62]. MDA reacts with TBA to form MDA-(TBA), a red adduct with a maximum absorbance at 532 nm (Beyotime Company, Jiangsu, China). For the MDA measurement, the cell lysates were added to the MDA detection solution and boiled for 15 min, followed by centrifugation at 1000 *g* for 10 min. Then, the supernatant was used to examine the relative MDA units using a Tecan Safire 5 microplate reader at a wavelength of 532 nm. Total SOD (T-SOD) activity was detected using SOD assay kits according to the manufacturer’s instructions (Beyotime Company, Jiangsu, China). Briefly, the cell lysates were mixed with nitroblue tetrazolium (NBT) and enzyme working solutions and incubated at 37 °C for 20 min. The absorbance of T-SOD was recorded at 560 nm using a Tecan Safire 5 microplate reader.

### Measurement of CAT

CAT activity was analysed according to the manufacturer’s instructions (Beyotime Company, Jiangsu, China). The samples were treated with excessive hydrogen peroxide and incubated for 5 min. Then the hydrogen peroxide not decomposed by CAT was coupled with a substrate to produce N-4-antipyryl-3-chloro-5-sulfonate-p-benzoquinone-monoimine after peroxidase treatment, which has an absorption maximum of 520 nm. The CAT unit was defined as the amount of enzyme that catalysed 1 μM of H_2_O_2_ to H_2_O and O_2_ per min at 25 °C.

### ROS assay

The concentration of ROS was fluorometrically monitored using DCFH-DA. The cells were treated with MEK inhibitor PD980025 (Sigma, USA) for 24 h prior to *M. ovipneumoniae* infection at an MOI of 100 through the application of the bacterial cells on the apical surface, followed by incubation at 37 °C overnight in 24-well plates. After the membranes were washed three times with PBS, DCFH-DA was diluted in fresh phenol red-free DMEM to a final concentration of 5 μM and incubated with the cells at 37 °C for 20 min. The chemicals were then removed, and the cells were washed three times. Relative ROS units were determined using a Tecan Safire 5 microplate reader (λex 485 nm and λem 530 nm). Changes in the ROS concentration were expressed as a percentage over the control.

### Immunoblotting analysis

Whole cell extracts were prepared by homogenizing cells in lysis buffer (50 mM Tris–HCl, pH 7.5, 5 mM EDTA, 150 mM NaCl, and 0.5 % NP-40) for 60 min on ice. The soluble protein concentration was measured with Bio-Rad Protein Assay (Bio-Rad Laboratories, Richmond, CA, USA). The resulting clarified lysates (100 μg) were separated using 8 % or 10 % sodium dodecyl sulphate (SDS)-polyacrylamide gel (SDS-PAGE) and transferred to nitrocellulose membranes for immunoblotting assay against antigen-specific antibodies. The membrane was blocked with 5 % fat-free dry milk in PBS containing 0.2 % Tween-20, and probed with antibodies against the protein of interest. The antibodies used in the present study included rabbit anti-SOD2, rabbit anti-RRAS, rabbit anti-RAF1, rabbit anti-ERK1/2, rabbit anti-BAK, rabbit anti-BAD, rabbit anti-BCL2, rabbit anti-Caspase 3, rabbit anti-Caspase 9, rabbit anti-Cytochrome c (Cyt-C) (Proteintech Group, Campbell Park, Chicago, USA), rabbit anti-phosphorylated Raf1 (p-Raf1), rabbit anti-MEK1/2, rabbit anti-phosphorylated MEK1/MEK2 (p-MEK1/2), rabbit anti-phosphorylated ERK1/2 (p-ERK1/2) (Signalway Antibody, MD, USA), rabbit anti-GSS (ABGENT, San Diego, USA) and mouse anti-β-actin. These primary antibodies were applied at a dilution of 1:1000. Following extensive washing, the membranes were incubated with an appropriate HRP-labelled secondary antibody. The blots were then developed using the enhanced Western Bright ECL reagent (Advansta, Menlo Park, CA, United States). The levels of protein expression were semi-quantified by optical densitometry using ImageJ Software version 1.46 (http://rsb.info.nih.gov/ij/). The ratio between the net intensity of each sample divided by the β-actin internal control was calculated as a densitometric arbitrary unit (A.U.), which served as an index of the relative expression of the protein of interest.

### Statistical analysis

The data were obtained from at least three independent experiments for each experimental condition and presented as the means ± standard deviation (SD). The statistical significance was analysed using one-way ANOVA, followed by Tukey’s multiple comparison test.

## Additional file

Additional file 1: Original images of immunoblots for Figure 4B and Figure 5A. (PDF 860 kb)

## Abbreviations

A.U.: Arbitrary unit
ALI: Air-liquid interface
BEGM: Bronchial epithelial cell Growth Medium
CAT: Catalase
CCU: Colour change unit
Cyt-C: Cytochrome C
DCFH-DA: 2′,7′-dichlorofluorescein diacetate
ERK: Extracellular signal-regulated kinase
GSS: Glutathione synthetase
LDH: Lactate dehydrogenase
MAPKs: Mitogen-activated protein kinases
MDA: Methane dicarboxylic aldehyde
MMP: Mitochondrial membrane potential
MO: *M. ovipneumoniae*/ *Mycoplasma ovipneumoniae*
MOI: Multiplicity of infection
NAC: N-acetyl-L-cysteine
NBT: Nitroblue tetrazolium
p-ERK1/2: phosphorylated ERK1/2
p-MEK1/2: phosphorylated MEK1/MEK2
p-Raf1: Phosphorylated Raf1
ROS: Reactive oxygen species
SD: Standard deviation
SDS: Sodium dodecyl sulfate
SDS-PAGE: (SDS)-polyacrylamide gel
SOD: Superoxide dismutase
TBA: Thiobarbituric acid
TLR: Toll-like receptor
T-SOD: Total SOD
USG: Ultroser G
