# Supplementary material for: Mycoplasma ovipneumoniae induces sheep airway epithelial cell apoptosis through an ERK signalling-mediated mitochondria pathway
Source: BMC Microbiol. 2016 Sep 23;16:222. doi: 10.1186/s12866-016-0842-0 (PMC5035462; doi:10.1186/s12866-016-0842-0)
Supplement: Additional file 1: — Original images of immunoblots for Figure 4B and Figure 5A. (PDF 860 kb) [file 12866_2016_842_MOESM1_ESM.pdf]

Supplementary files

Supplementary file 1. Original images of immunoblots for Figure 4B and Figure 5A.

Data 1: Fig. 4B Original image for immunoblot of Actin

Loading 1 2 3 4 5 6 7 8 9 10

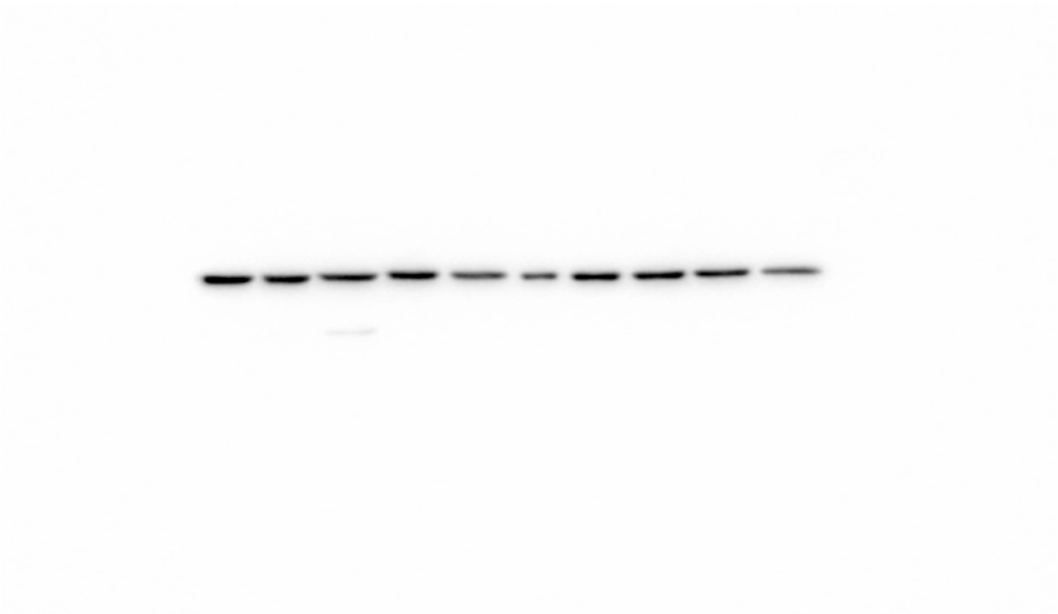

1. MO
2. MO+NAC
3. MO+L-NMNIA
4. MO+PD980025
5. MO+SP600125
6. L-NMNIA
7. MOCK
8. NAC
9. PD980025
10. SP600125

Data 2: Fig. 4B Original image for immunoblot of ERK

Loading 1 2 3 4 5 6 7 8 9 10

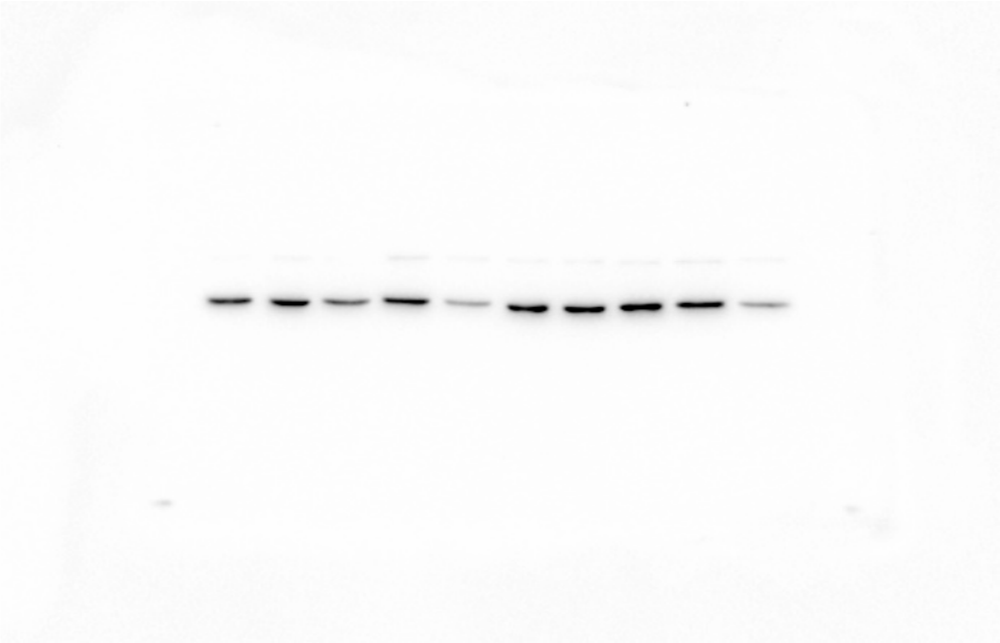

1. MO
2. MO+NAC
3. MO+L-NMNIA
4. MO+PD980025
5. MO+SP600125
6. L-NMNIA
7. MOCK
8. NAC
9. PD980025
10. SP600125

Data 3: Fig. 4B Original image for immunoblot of MEK

Loading 1 2 3 4 5 6 7 8 9 10

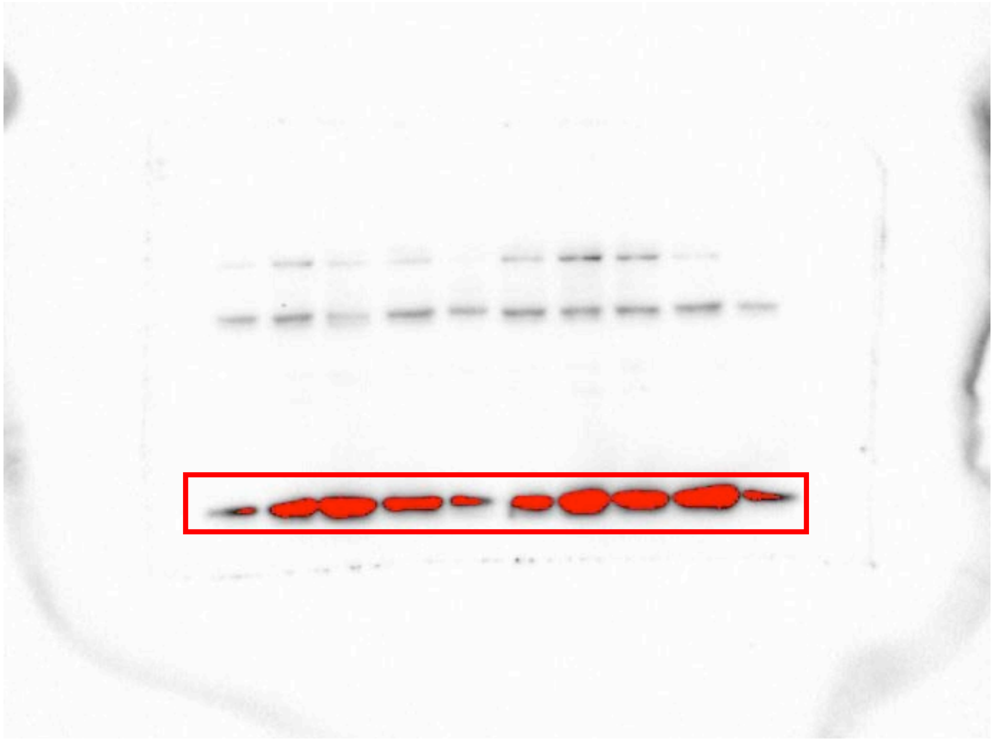

1. MO
2. MO+NAC
3. MO+L-NMNIA
4. MO+PD980025
5. MO+SP600125
6. L-NMNIA
7. MOCK
8. NAC
9. PD980025
10. SP600125

Data 4: Fig. 4B Original image for immunoblot of phos-ERK

Loading 1 2 3 4 5 6 7 8 9 10

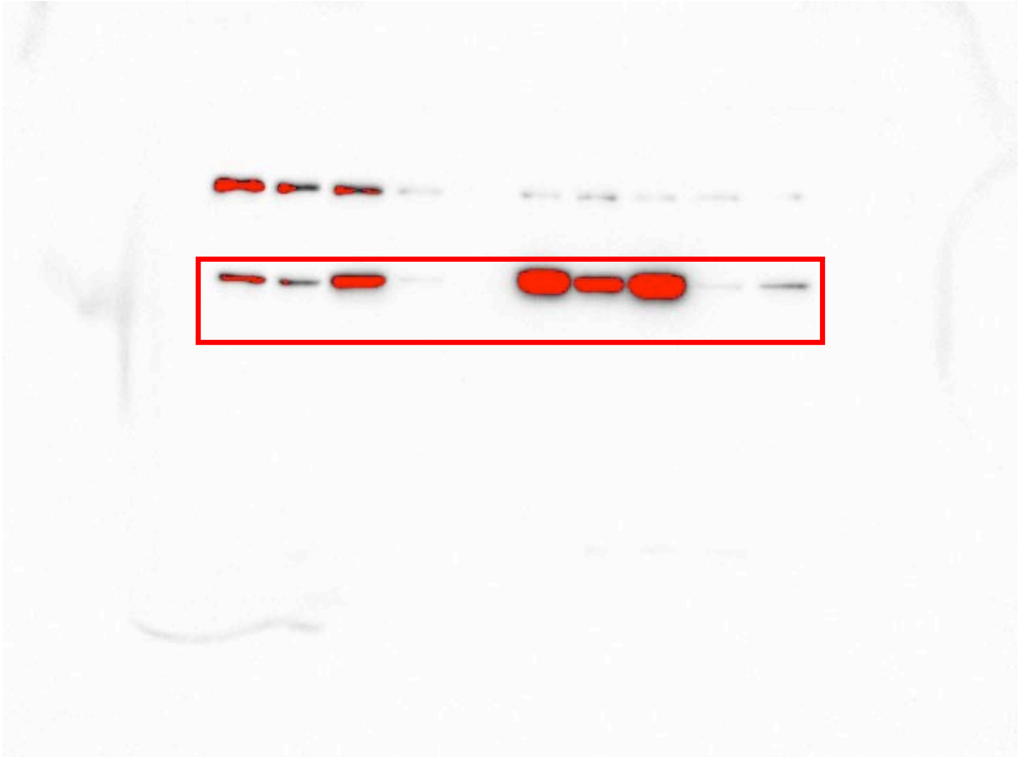

1. MO
2. MO+NAC
3. MO+L-NMNIA
4. MO+PD980025
5. MO+SP600125
6. L-NMNIA
7. MOCK
8. NAC
9. PD980025
10. SP600125

Data 5: Fig. 4B Original image for immunoblot of phos-MEK

Loading 1 2 3 4 5 6 7 8 9 10

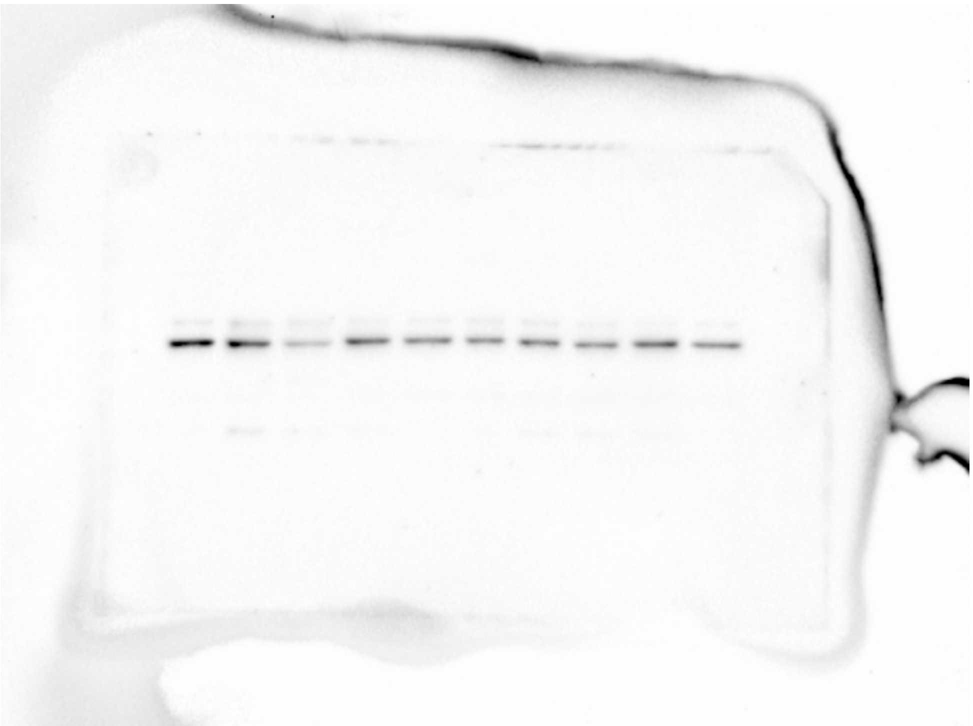

1. MO
2. MO+NAC
3. MO+L-NMNIA
4. MO+PD980025
5. MO+SP600125
6. L-NMNIA
7. MOCK
8. NAC
9. PD980025
10. SP600125

Data 6: Fig. 4B Original image for immunoblot of phos-RAF

Loading 1 2 3 4 5 6 7 8 9 10

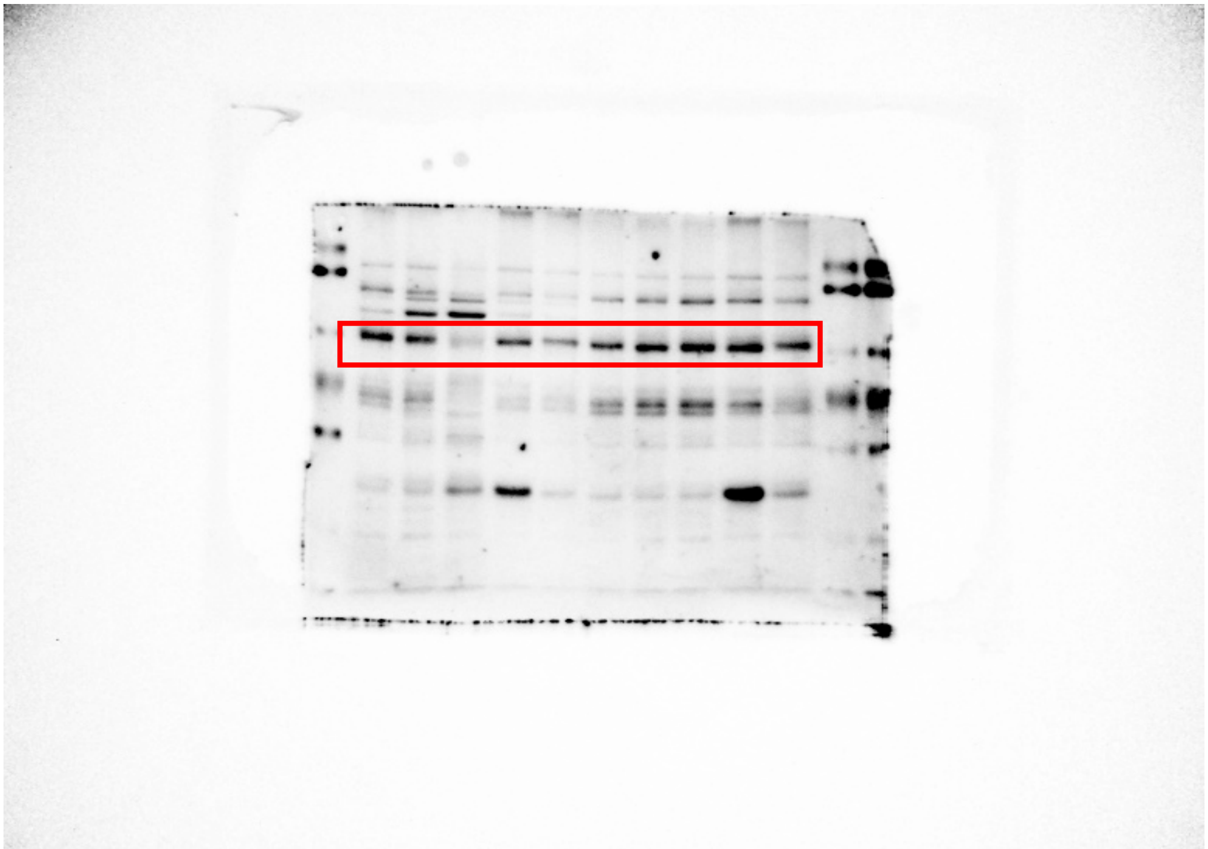

1. MO
2. MO+NAC
3. MO+L-NMNIA
4. MO+PD980025
5. MO+SP600125
6. L-NMNIA
7. MOCK
8. NAC
9. PD980025
10. SP600125

Data 7: Fig. 4B Original image for immunoblot of RAF

Loading 1 2 3 4 5 6 7 8 9 10

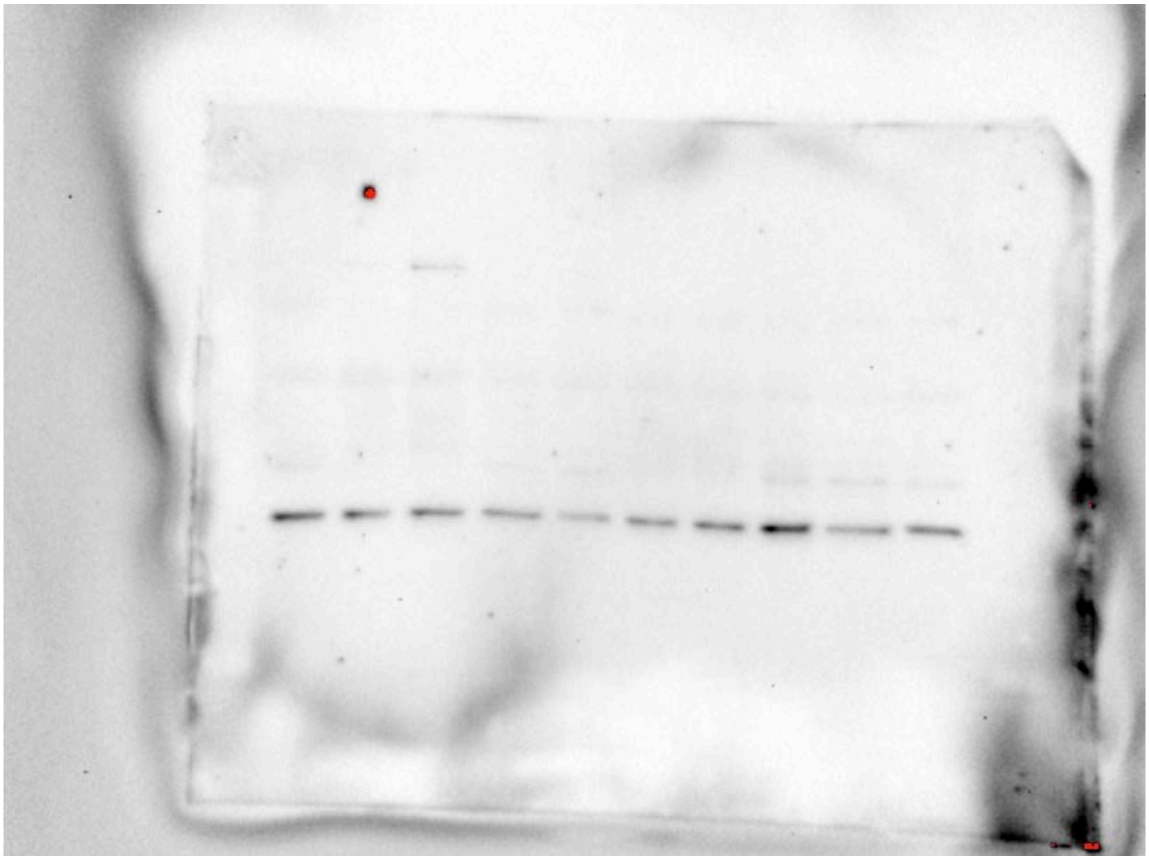

1. MO
2. MO+NAC
3. MO+L-NMNIA
4. MO+PD980025
5. MO+SP600125
6. L-NMNIA
7. MOCK
8. NAC
9. PD980025
10. SP600125

Data 8: Fig. 4B Original image for immunoblot of RAS

Loading 1 2 3 4 5 6 7 8 9 10

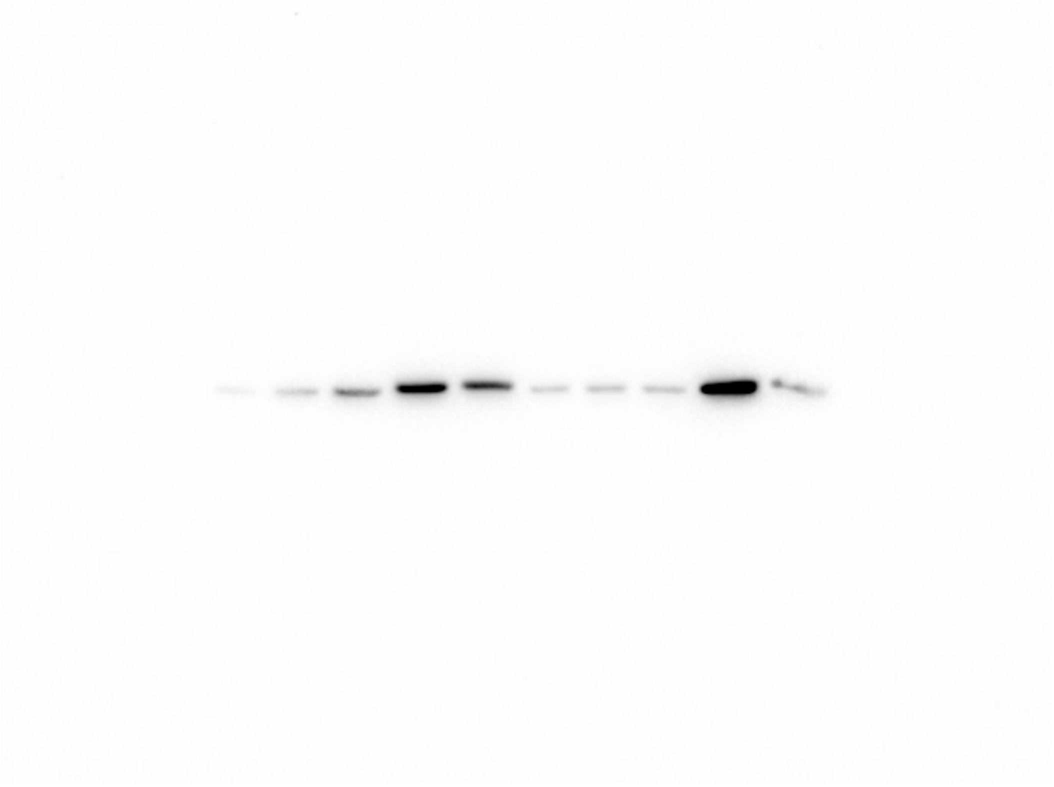

1. MO
2. MO+NAC
3. MO+L-NMNIA
4. MO+PD980025
5. MO+SP600125
6. L-NMNIA
7. MOCK
8. NAC
9. PD980025
10. SP600125

Data 9: Fig. 5A Original image for immunoblot of BAD

Loading 1 2 3 4 5 6 7 8 9 10

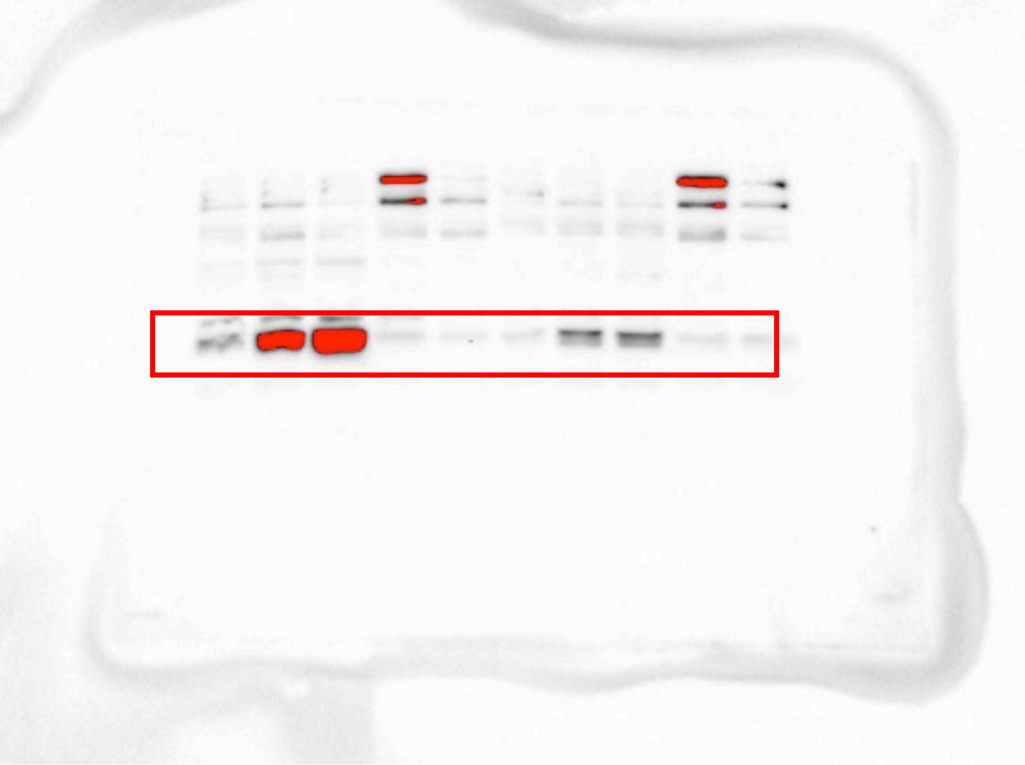

1. MO
2. MO+NAC
3. MO+L-NMNIA
4. MO+PD980025
5. MO+SP600125
6. L-NMNIA
7. MOCK
8. NAC
9. PD980025
10. SP600125

Data 10: Fig. 5A Original image for immunoblot of BAK

Loading 1 2 3 4 5 6 7 8 9 10

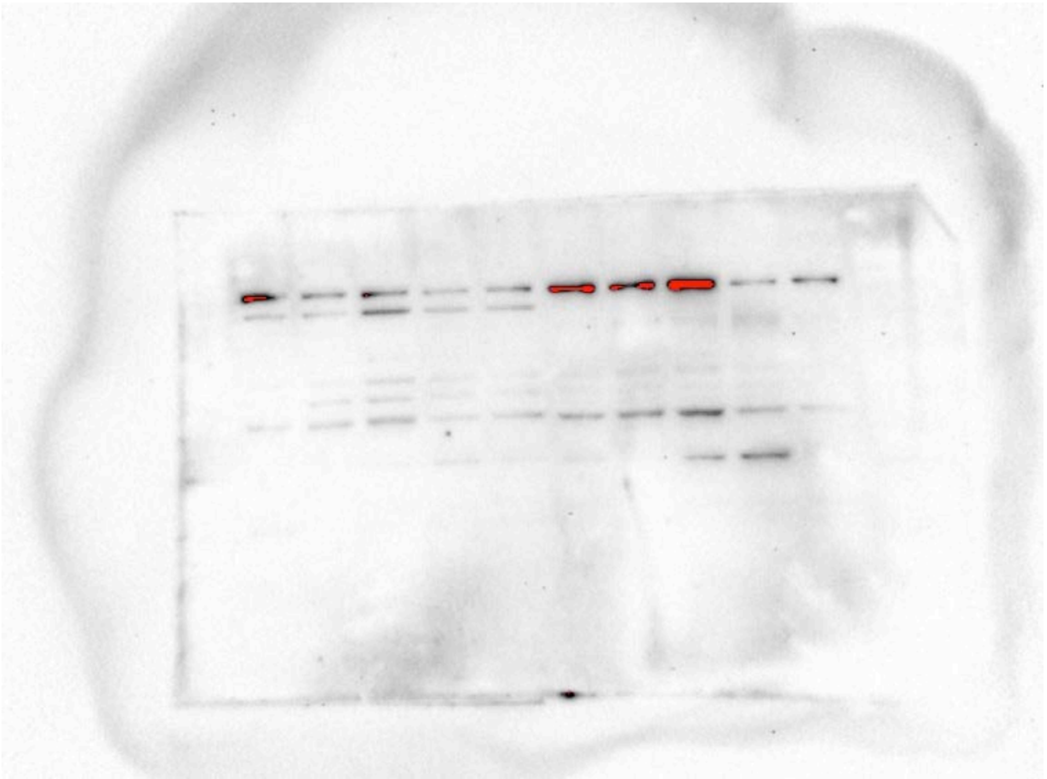

1. MO
2. MO+NAC
3. MO+L-NMNIA
4. MO+PD980025
5. MO+SP600125
6. L-NMNIA
7. MOCK
8. NAC
9. PD980025
10. SP600125

Data 11: Fig. 5A Original image for immunoblot of BCL-2

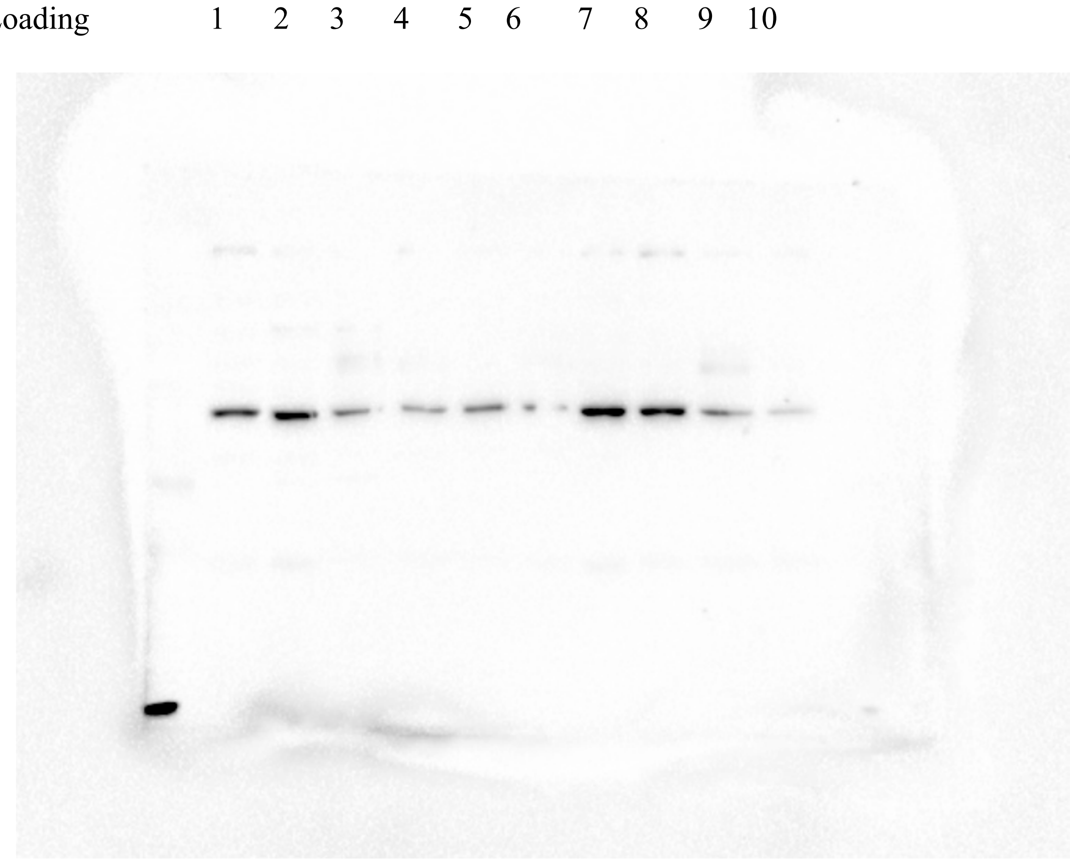

1. MO
2. MO+NAC
3. MO+L-NMNIA
4. MO+PD980025
5. MO+SP600125
6. L-NMNIA
7. MOCK
8. NAC
9. PD980025
10. SP600125

Data 12: Fig. 5A Original image for immunoblot of BCL-xl

Loading 1 2 3 4 5 6 7 8 9 10

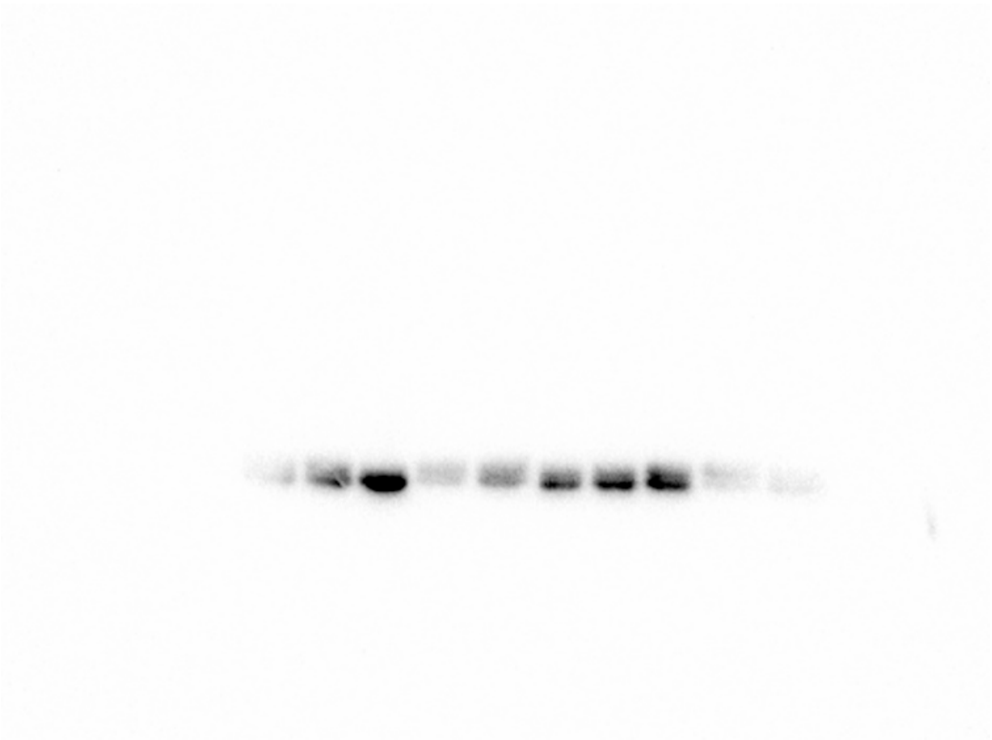

1. MO
2. MO+NAC
3. MO+L-NMNIA
4. MO+PD980025
5. MO+SP600125
6. L-NMNIA
7. MOCK
8. NAC
9. PD980025
10. SP600125
